# Modelling the Effect of Diet Composition on Enteric Methane Emissions across Sheep, Beef Cattle and Dairy Cows

**DOI:** 10.3390/ani6090054

**Published:** 2016-09-08

**Authors:** Matt Bell, Richard Eckard, Peter J. Moate, Tianhai Yan

**Affiliations:** 1 School of Biosciences, The University of Nottingham, Sutton Bonington, Loughborough LE12 5RD, UK; 2 Melbourne School of Land and Environment, University of Melbourne, Victoria 3010, Australia; rjeckard@unimelb.edu.au; 3 Department of Economic Development, Jobs, Transport and Resources, 1301 Hazeldean Road, Ellinbank, Victoria 3820, Australia; peter.moate@ecodev.vic.gov.au; 4 Agr-Food and Biosciences Institute, Hillsborough T26 6DR, UK; tianhai.yan@afbini.gov.uk

**Keywords:** sheep, cattle, enteric methane, diet, prediction, modelling

## Abstract

**Simple Summary:**

Enteric methane emissions produced by ruminant livestock has gained global interest due to methane being a potent greenhouse gas and ruminants being a significant source of emissions. In the absence of measurements, prediction models can facilitate the estimation of enteric methane emissions from ruminant livestock and aid investigation of mitigation options. This study developed a practical method using feed analysis information for predicting enteric methane emissions from sheep, beef cattle and dairy cows fed diets encompassing a wide range of nutrient concentrations.

**Abstract:**

Enteric methane (CH_4_) is a by-product from fermentation of feed consumed by ruminants, which represents a nutritional loss and is also considered a contributor to climate change. The aim of this research was to use individual animal data from 17 published experiments that included sheep (*n* = 288), beef cattle (*n* = 71) and dairy cows (*n* = 284) to develop an empirical model to describe enteric CH_4_ emissions from both cattle and sheep, and then evaluate the model alongside equations from the literature. Data were obtained from studies in the United Kingdom (UK) and Australia, which measured enteric CH_4_ emissions from individual animals in calorimeters. Animals were either fed solely forage or a mixed ration of forage with a compound feed. The feed intake of sheep was restricted to a maintenance amount of 875 g of DM per day (maintenance level), whereas beef cattle and dairy cows were fed to meet their metabolizable energy (ME) requirement (i.e., production level). A linear mixed model approach was used to develop a multiple linear regression model to predict an individual animal’s CH_4_ yield (g CH_4_/kg dry matter intake) from the composition of its diet. The diet components that had significant effects on CH_4_ yield were digestible organic matter (DOMD), ether extract (EE) (both g/kg DM) and feeding level above maintenance intake: CH_4_ (g/kg DM intake) = 0.046 (±0.001) × DOMD − 0.113 (±0.023) × EE − 2.47 (±0.29) × (feeding level − 1), with concordance correlation coefficient (*CCC*) = 0.655 and RMSPE = 14.0%. The predictive ability of the model developed was as reliable as other models assessed from the literature. These components can be used to predict effects of diet composition on enteric CH_4_ yield from sheep, beef and dairy cattle from feed analysis information.

## 1. Introduction

Abatement of CH_4_ emissions from livestock has gained importance due to the association between greenhouse gas (GHG) concentrations in the atmosphere and global climate change. Dietary manipulation is a mitigation option that can be applied immediately [1]. To facilitate the estimation of enteric CH_4_ emissions from ruminant livestock and investigate mitigation options, a number of dynamic mechanistic and empirical prediction models have been developed over a number of years. In the absence of a direct, easy and accurate measure of dietary energy lost in the form of CH_4_, prediction models can offer some understanding of what influences CH_4_ production and provide a tool for assessing mitigation options. Typically, prediction models have been developed based on either an empirical or dynamic mechanistic approach. Several empirical equations have been developed and evaluated [2,3,4] and such models can offer a more practical method for estimating CH_4_ emissions compared to dynamic mechanistic models. Dynamic mechanistic methods are more complex and use mathematical descriptions of rumen fermentation. Such models are based on knowledge of the underlying mechanisms, and often are based on inputs of the rate of rumen fermentation, especially the pattern and rate of production of acetic and propionic acids and the rate of production of hydrogen in the rumen. However, this approach has been found to show a great degree of adaptability across diet types and feed intakes [2,5]. 

For the purpose of evaluating mitigation options for different livestock species and further development of national inventory methodology, a practical prediction equation that is accurate and precise, and can reliably be applied across livestock species and levels of feed intake would be useful for national inventories. The lack of a robust prediction of CH_4_ across species is partly due to availability of input variables required, the range of values from which equations were developed, and the inability to model degradability of feeds and/or passage rate of feed consumed on enteric CH_4_ in different livestock species. There are few prediction models developed for both cattle and sheep [6,7], with the widely used model of Blaxter and Clapperton [6] being found to be unsuitable for ruminants with a DM intake greater than 15 kg/day [8], a level of intake that is commonly greatly exceeded in modern high yielding dairy cows [9]. 

The objectives of the current study were thus to (1) develop a prediction model across sheep, beef cattle and dairy cows to quantify the effect of diet composition on enteric CH_4_ yield (g/kg DM intake) and (2) evaluate the predictive ability of the developed model alongside those of other published equations. 

## 2. Materials and Methods

The current study utilized data from sheep, beef cattle and dairy cows studies performed in the UK and Australia where detailed information on a wide range of diet composition was available, and CH_4_ was measured via respiration calorimetry. There were individual records for 288 sheep, 71 beef cattle and 252 dairy cows obtained and included in the current analysis, with two-thirds of the data being randomly selected for model development and the remaining one-third of the data being used for model evaluation as described below.

### 2.1. Data

Data on animal measures (DM intake, live weight, and milk yield where applicable), nutrient composition of feed, and CH_4_ production (g/day) and CH_4_ yield (g/kg DM intake) for individual animals were obtained from a total of 17 experiments carried out at the Rowett Feedingstuffs Evaluation Unit (Rowett), at the Agri-Food and Biosciences Institute (AFBI), and at Ellinbank Research Centre (Ellinbank). The Rowett and AFBI institutes are in the UK, while Ellinbank is in Australia, and experiments were conducted between the years of 1970 to 2008. Data used for model development are summarized in Table 1, while data used for model evaluation are summarized in Table 2. The data obtained from UK institutes (in the 1970s and 1980s) has contributed to the development of the UK metabolizable energy (ME) feeding system [10] currently in use. The focus of these studies was to assess energy values of feeds rather than CH_4_ emissions of animals. Results from these feed trials were published in a series of reports [11,12,13,14,15]. These data have been used by others as well [16] to assess CH_4_ emissions from individual sheep fed single feeds. In comparison, the current study used Rowett Institute data from sheep fed mixed rations of forage and a compound feed rather than a single feed.

At the Rowett Institute, sheep were fed a maintenance diet and measurements were made of energy intake and of losses of energy in faeces, in urine, and as enteric CH_4_. Data from these trials were extracted from the Third Report of the Rowett Feedingstuffs Evaluation Unit [13]. In this work, adult wether sheep were offered 875 g of DM per day for a period of 28 days. For the first 16 days, sheep were in metabolism stalls for adaptation to the diet; for the following 12 days, individual sheep were housed in closed-circuit calorimeters (chamber) and energy losses including CH_4_ were measured during the final 2 days. A total of twenty-four compound feeds, blended from thirty-five ingredients, were evaluated. Each compound feed was included in six mixed rations with grass silage or grass hay at ratios of 75:25 (low forage), 50:50 (medium forage) and 25:75 (high forage), which were fed twice daily. Two sheep were fed each ration (forage x ratio), and thus twelve sheep were fed each compound feed. This provided 288 individual sheep records i.e., 2 × 3 diet ratios × 2 forage types × twenty-four compound feeds. Diets were designed to encompass a wide range of crude protein (92 to 192 g/kg DM), EE (17 to 66 g/kg DM) and crude fibre (116 to 322 g/kg DM) concentrations. See the Third Report of the Rowett Feedingstuffs Evaluation Unit [13] for a description of feed analysis.

There were 71 individual beef cattle records (from 31 beef steers) obtained from four energy metabolism studies conducted at AFBI between 1993 and 1999 [18]. The animals used were of various ages (18 to 21 months) and breeds (Friesian, Aberdeen Angus, Simmental and Charolais). The live weight of steers ranged from 363 to 627 kg and DM intake ranged from 4.7 to 10.2 kg/day (Table 1). Steers were offered either grass silage alone as a sole diet (*n* = 36), or a mixture of grass silage and concentrates (*n* = 35) at production feeding levels and fed once per day. In the latter situation, the concentrates were offered either mixed as a complete diet with silage, or separately. The proportion of grass silage in diets ranged from 0.29 to 1 with a mean of 0.81 (s.d. 0.23). The grass silages encompassed primary growth and first and second re-growth material, harvested from perennial ryegrass swards. The harvested grass was either unwilted or wilted before ensiling, and ensiled with or without application of silage additives. The concentrates used were based on barley, maize, wheat, soyabean meal, citrus pulp and mineral and vitamin supplement. Prior to commencing energy metabolism measurements, all steers were offered the experimental diets for at least 3 weeks in group-housed pens before measurement of energy metabolism data (described below).

The dairy cow dataset from AFBI consisted of 222 individual lactating dairy cow records (from 110 cows) of different breeds (Holstein-Friesian, Norwegian Red, and Jersey-Holstein) in 10 energy metabolism studies conducted between 1993 and 2007 [19]. The cows used covered wide ranges of milk yield potential, lactation number (1–9), stage of lactation (early to late), and live weight (385–733 kg). Milk yield during energy metabolism measurements ranged from 3.1 to 49.1 kg/day and dry matter intake from 7.5 to 24.5 kg/day (Table 1). All cows were offered grass silage-based diets (*n* = 222) ad libitum and fed once per day. A total of 35 cows were offered grass silage as the sole diet. The proportion of grass silage in diets ranged from 0.25 to 1 with a mean of 0.55 (s.d. 0.21). All silage was harvested from perennial ryegrass swards. The grass silages encompassed primary growth and first and second regrowth material. The harvested grass was either unwilted or wilted before ensiling, and ensiled with or without application of silage additives. The concentrates used in each of the studies included a vitamin-mineral supplement and combinations of the following ingredients: cereal grains (barley, wheat, or maize), by-products (maize gluten meal, molassed or unmolassed sugar-beet pulp, citrus pulp, or molasses), and protein supplements (fish meal, soyabean meal, or rapeseed meal). The concentrate portion of the diet was offered either in a complete diet mixed with grass silage or as a separate feed. Prior to commencing energy metabolism measurements, all cows were offered experimental diets for at least 3 weeks in group-housed pens in cubicle accommodation. In the metabolism unit at AFBI, each cow was housed for 8 days with total collection of faeces and urine during the final 6 days. Immediately after completion of the period of faeces and urine collection, each cow was transferred to an indirect open-circuit calorimeter. Cows remained in calorimeters for 3 days with measurement of gaseous exchange over the final 48 h period.

At Ellinbank, lactating Holstein-Friesian cows were used in an extended lactation experiment [20] over a two-year period (i.e., breeding was delayed until approximately 450 days in milk, with a target lactation length of 670 days). During the lactation, there were four three-week experimental periods using 16 cows, comprising time spent in the paddock, metabolism stalls and then calorimetric chambers. Within pairs, eight cows were allocated to either a forage only diet (fresh cut pasture during periods 1 and 3 (spring) or grass silage/alfalfa hay during periods 2 and 4 (autumn) for consecutive years) or to the same forage supplemented with grain (Grain group). A total of 62 individual lactating dairy cow records were obtained from the study period, with two records missing. Prior to commencing energy metabolism measurements, all cows were offered experimental diets for at least 2 weeks. Cows spent 4 days in metabolism stalls followed by 3 days in a chamber for CH_4_ measurements. When cows entered the metabolism stalls, they were, on average, 110 ± 12 (mean ± SD, Period 1), 270 ± 12 (Period 2), 450 ± 14 (Period 3) and 560 ± 12 (Period 4) days in milk. During energy metabolism measurements the live weight of cows ranged from 416 to 687 kg, milk yield ranged from 4.5 to 34.8 kg/day and dry matter intake from 11.8 to 19.7 kg/day (Table 1 and Table 2). Diets were fed twice daily. Fresh cut pasture comprised a mixture of diploid and tetraploid ryegrass hybrids between perennial ryegrass (*Lolium perenne* L.) and Italian ryegrass (*Lolium multiflorum* L.), designated *Lolium hybridum*. The pasture provided approximately 150 MJ ME/cow/day. The grass silage was made from the above pasture and alfalfa hay (*Medicago sativa* L.) to provide the same amount of ME. The Grain group received 4.4 to 5.0 kg DM of cereal grain per day (average concentration per kg DM: 177 g crude protein, 120 g NDF, 19 g EE and 14.4 MJ ME), which increased ME intake to approximately 205 MJ/cow/day.

### 2.2. Data Used to Develop and Evaluate Model Predictions

Two-thirds of the individual records for sheep at the Rowett Institute, beef cattle and dairy cows at AFBI and dairy cows at Ellinbank were randomly selected for CH_4_ prediction model development. The remaining third of data were used to evaluate the predictive power of the developed model alongside those of other published equations. This provided individual records from 192 sheep, 47 beef cattle and 189 dairy cows for model development (Table 1) and individual records from 96 sheep, 24 beef cattle and 95 dairy cows for model evaluation (Table 2). The random selection of data used for model development was repeated five times to minimise the chance of bias in data selected for model development and validation, as recommended by Rodriguez et al. [21]. The data and results presented were therefore average values for the five random subsets of data used for model development and validation.

### 2.3. Statistical Analysis

Data were analysed using Genstat Version 16.1 (Lawes Agricultural Trust, London, UK) [22]. A linear mixed model (Equation (1)) was used to assess the effect of explanatory variables (diet components) on enteric CH_4_ yield per individual animal across animal species:

*Y_i_* = b_1_x_1_ + b_2_x_2_ … b_n_x_n_ + S*_i_* + E*_i_*, (1)

where *Y_i_* is the dependent variable of CH_4_ yield in g/kg DM intake*;* b_1_x_1_ to b_n_x_n_ = linear regression of *Y* on x-variables (fixed effects); and S*_i_* = random effect of experiment; E*_i_* = residual error within experiment. The residual within-experiment variance was allowed to differ between experiments.

Each diet component was first analysed for its relationship with CH_4_ yield in a univariate analysis and correlation coefficients between diet components were calculated. All the most significant variables (with *p <* 0.25) from the univariate analyses were added initially to a linear mixed model and only those variables that made a significant (*p <* 0.05) additional contribution when fitted last were retained [23]. This traditional approach to statistical model building minimizes the number of variables to ensure that the resulting model is numerically stable. Interactions between diet components and livestock type were tested for inclusion in the model, but livestock type and its interaction with diet components were not found to be significant and therefore not included in the final model.

### 2.4. Diet Components

Diet components evaluated in the analysis were: digestible organic matter in the total DM (DOMD), cellulose, hemicellulose, lignin, starch, sugar, neutral detergent fibre, acid detergent fibre, crude fibre, EE (oil), crude protein, ash (all g/kg DM), and the energy contents of gross (GE), digestible (DE) and ME (all MJ/kg DM). The non-fibre carbohydrate (1000 – (neutral detergent fibre + ash + crude protein + EE)) concentration of feed was calculated and included in the analysis. The chemical compositions of feeds were measured in each experiment unless otherwise stated. For full details of feed analysis procedures used in the experiments at Rowett Institute, see Wainman et al. [13], for Ellinbank Research Centre, see Williams et al. [20], and for AFBI, see Mayne and Gordon [24]. The DOMD at the Rowett Institute was calculated by multiplying the organic matter content of the feed by the digestibility of organic matter (OMD), where OMD was measured in sheep fed at maintenance, but not in the other experiments studied using beef cattle and dairy cows. In beef cattle and dairy cows fed at their production intake level, the DOMD was estimated from measured ME concentrations of feed using data in Third Report of the Rowett Feedingstuffs Evaluation Unit [13] to produce Equation (2):

DOMD (g/kg DM) = 472.49 × ln (ME) − 437.69.(2)

This assumes a curvilinear relationship between ME concentration of feed and DOMD [25], rather than a linear relationship as proposed by the AFRC [17], i.e., (ME/0.16) × 10. In addition to these diet components, the effect of feeding level on enteric CH_4_ yield was included in the analysis and calculated as multiples of ME intake over maintenance energy requirements of the animal calculated from AFRC [17]. The feeding level variable was expressed as multiples of ME intake above maintenance intake, i.e., the effect of feeding level on enteric CH_4_ is zero at the maintenance intake level [26]. 

### 2.5. Model Evaluation

Pearson correlation coefficient (*r*) was used to test the association between DM intake and daily CH_4_ emissions, and the correlation between explanatory variables for CH_4_ yield. The coefficient *r* was used to measure how far observations deviated from the best-fit line. Coefficient *r* was multiplied by Lin’s bias correction factor (*C_b_*), which measures how far the best-fit line deviates from the 45° line through the origin, in order to derive the concordance correlation coefficient (*CCC*) [27]. The location shift (ν) and scale shift (μ) values were calculated to compare the means and standard deviations for observed and predicted CH_4_ yields, respectively. 

The coefficient *CCC* was used to test the association between pairs of sheep for ME of feed (MJ/kg DM) and CH_4_ yield (MJ/kg DM) at the Rowett Institute. The coefficient *CCC* was also used to test the association between observed and predicted CH_4_ yields. Observed (*O_i_*) and predicted (*P_i_*) CH_4_ yields were also compared using Equation (3) and the mean square prediction error (MSPE) for all observations (*n*):

(3) $$\mathrm{MSPE}=\sum_{i=1}^{n}{\left(Oi-Pi\right)}^{2}/n$$

The square root of the MSPE (RMSPE), expressed as a percentage of the observed mean CH_4_ emissions, gives an indication of the overall prediction error. In the analysis, the MSPE was separated into error due to overall mean bias (ER), error due to deviation of the regression slope from unity (ECT), and random error (ED) [28]. The MSPE was adjusted for the random effect of experiment. 

Several linear and nonlinear prediction equations for CH_4_ emissions developed using sheep and/or cattle were obtained from the literature and compared to the prediction model developed in the current study. The prediction equations from the literature (Table 3) that were selected have been evaluated and recommended in other evaluation studies [2,3], and can also predict CH_4_ emissions for feed intakes of 0.875 kg DM/day and above [8] i.e., a wide range of DM intakes. 

## 3. Results

**Figure 1:**
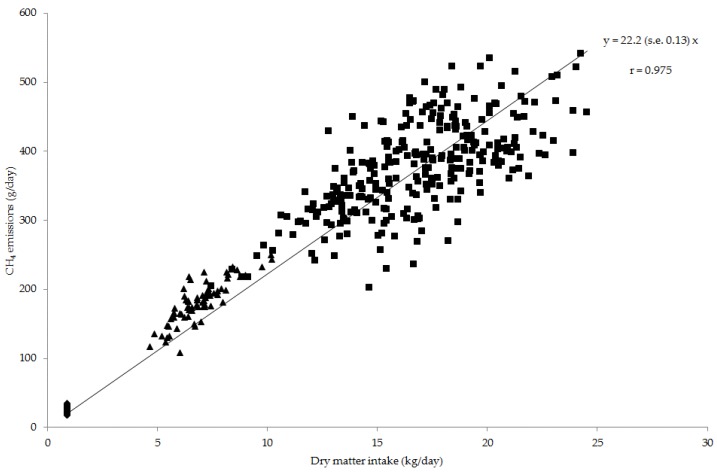
Observed dry matter intake and CH_4_ emissions per day for sheep (♦; *n* = 288), beef cattle (▲; *n* = 71) and dairy cows (■; *n* = 284) included in the analysis. The line of best-fit through the origin across all values is shown with the Pearson correlation coefficient (*r*).

Across sheep, beef cattle and dairy cows, the daily CH_4_ emissions were positively related to DM intake (22.2 (s.e. 0.13) g/kg DM; Figure 1, *r* = 0.975, *p* < 0.001). For cattle fed at their production intake level, there was a high and positive correlation between DM intake and daily CH_4_ emissions for beef cattle (*r* = 0.837, *p* < 0.001) and dairy cows (*r* = 0.668, *p* < 0.001).

In the ME work at the Rowett Institute, each pair of sheep was studied at the same time while being fed the same diet, but in separate calorimeters, which allows variation among paired sheep on the same diet to be compared. For pairs of sheep fed the same diet, the bias correction factor of best-fit line to the 45° line through the origin was high for both ME content (*C_b_* = 0.999) and CH_4_ yield (*C_b_* = 0.996), whereas, the correlation and *CCC* values were high for ME content (*r* = 0.927, *p* < 0.001; *CCC* = 0.926) and low for CH_4_ yield (*r* = 0.219, *p* < 0.001; *CCC* = 0.218).

The coefficient of variation in CH_4_ yield between animals was lowest for beef cattle (9.8%) and highest for dairy cows (15.0%), with the observed mean CH_4_ yield being 29.3 ± 3.6 g/kg DM intake for sheep, 26.4 ± 2.6 g/kg DM intake for beef cattle and 22.6 ± 3.4 g/kg DM for dairy cows. 

### 3.1. Significant Diet Components

Across sheep, beef cattle and dairy cows, CH_4_ yield was positively related to DOMD and negatively related to EE and feeding level (all *p <* 0.001). The following prediction Equation (4) for CH_4_ yield was derived for sheep and cattle across diets and intake levels:

CH_4_ (g/kg DM intake) = 0.046 ± 0.001 × DOMD − 0.113 ± 0.023 × EE (both g/kg DM) − 2.47 ± 0.29 × (feeding level − 1) (*CCC* = 0.655; RMSPE = 14.0%). (4)

The regression coefficients for these significant effects on CH_4_ yield for sheep, beef cattle and dairy cows were 0.049 ± 0.006, 0.007 ± 0.010 and 0.003 ± 0.006 for DOMD, −0.185 ± 0.030, −0.112 ± 0.174, and −0.058 ± 0.052 for EE, −3.00 ± 1.38 and −2.06 ± 0.35 for feeding level of cattle, respectively. 

### 3.2. Evaluating Predictions

**Figure 2:**
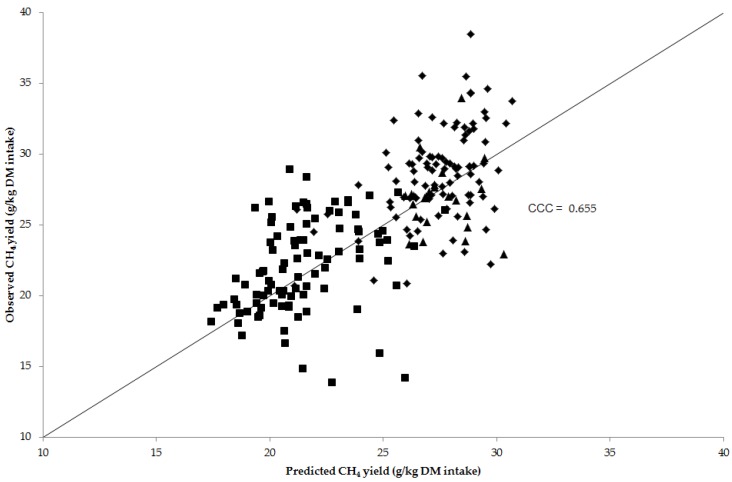
Predicted using Equation (4) and observed CH_4_ yield (g/kg dry matter (DM) intake) for sheep (♦; *n* = 96), beef cattle (▲; *n* = 24) and dairy cows (■; *n* = 95). The concordance correlation (*CCC*) is shown across species and the 45° line through the origin.

Predictions using Equation (4) and equations from the literature for CH_4_ yield from sheep fed at their maintenance intake and cattle fed at their production level were assessed against observed CH_4_ values (Table 4 and Figure 2).

The RMSPE prediction errors between observed values and predictions were less than 20% for several equations, which were Equation (4), Equations (7) to (9), and Equation (13). Overall, of the equations evaluated, Equation (4) across species and Equation 13 both had the lowest RMSPE of 14.0%. Within species, Equation (4) produced the lowest RMSPE when predicting beef cattle CH_4_ yield (11.1%), but also had a low and negative correlation coefficient (*r* = −0.044), *CCC* (−0.017), over-prediction of average CH_4_ yield (ν = −0.53) and lower standard deviation (μ = 2.05) compared to observed values. Whereas predictions of CH_4_ yield for sheep and dairy cows using Equation (4) had a similar RMSPE (13.7% and 15.4%), correlation coefficient (*r* = 0.456 and 0.413), *CCC* (0.321 and 0.303), under-prediction of average CH_4_ yield (ν = 0.62 and 0.37) and lower standard deviation (μ = 1.93 and 1.62) compared to observed values. 

The breakdown of MSPE showed that the largest proportion of error associated with predictions for Equation (4) across species, and Equations (7) to (9) and Equation (13) were ED (0.62 to 0.83). The predictions from these equations had high and positive correlation coefficients (ranging from *r* = 0.543 to 0.696) and high Lin’s bias correction factors (ranging from *C_b_* = 0.785 to 0.974) when compared with observed CH_4_ yield. This resulted in *CCC* values ranging from 0.477 for Equation (7) to 0.673 for Equation (13). In addition, Equation (4) across species, Equations (7) to (9) and Equation (13) all slightly under-predicted the average CH_4_ yield (ν ranged from 0.14 to 0.64) and had a lower standard deviation (μ ranged from 1.21 to 1.75) compared to observed values.

**Figure 3:**
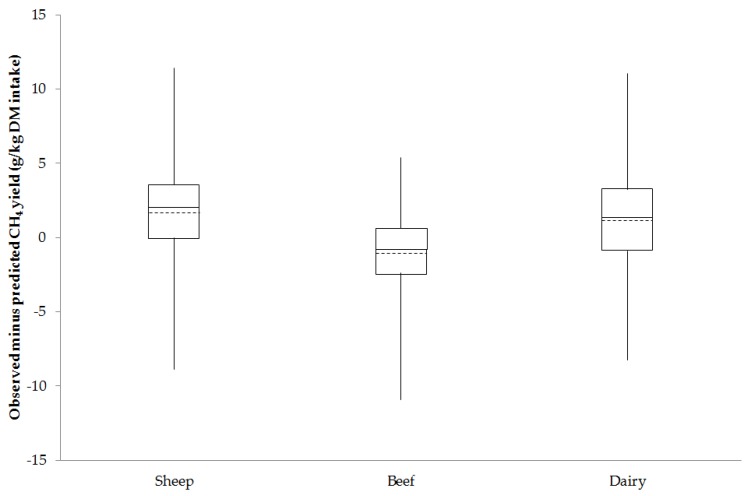
A box and whisker diagram showing the minimum, lower quartile, mean (- - -), median, upper quartile and maximum for observed minus predicted CH_4_ yield (g/kg dry matter (DM) intake) across sheep, beef cattle and dairy cow data.

After accounting for the significant fixed effects of DOMD, EE and feeding level on CH_4_ yield (Equation (4)) and random effects of experiments, there was still notable residual variation (difference between observed and predicted values) in CH_4_ emissions among cattle and sheep (Figure 3). Overall, sheep and cattle species were associated with a similar range of residual values, but beef cattle had a lower mean and median residual value compared to sheep and dairy cows.

## 4. Discussion

Other than studies by Blaxter and Clapperton [6] and Ramin and Huhtanen [7], the authors are unaware of another study that has developed an empirical prediction equation for use across sheep and cattle to predict CH_4_ yield using common feed analysis information. Furthermore, the prediction model in the current study was developed using diets encompassing a wide range in forage proportion (0.25 to 1), nutrient concentrations (i.e., 235 to 649 g NDF/kg DM, 92 to 251 g crude protein/kg DM, 17 to 64 g EE/kg DM and 9 to 14 MJ ME/kg DM) and CH_4_ yield (14 to 40 g/kg DM). This was made possible by combining data from different institutes and experiments using sheep and cattle fed a range of diets and wide range of nutrient concentrations. For example, the diets fed to sheep at the Rowett institute encompassed a wider range of EE concentrations (17 to 64 g/kg DM) compared to other data sets used in model development (26 to 63 g/kg DM). The results of this study suggest that the CH_4_ yield from sheep and cattle across feed intake levels can be predicted using a single equation that includes the diet components of DOMD, EE and feeding level (Equation (4)). Expressing CH_4_ emitted per unit intake i.e., CH_4_ yield (g CH_4_/kg DM intake) seemed appropriate when assessing the effect of diet on emissions given the high and positive correlation between DM intake and CH_4_ emissions across sheep and cattle (*r* = 0.98; Figure 1). It is recognised that DM intake has a large effect on enteric CH_4_ emissions from ruminants [7,25,31,32]. Furthermore, the significant input variables can be easily obtained from feed analysis information and therefore offer a practical and easy prediction of dietary energy lost as enteric CH_4_ from ruminants fed at different feeding levels. This also provides a means of investigating feed composition abatement options for ruminants fed a range of diets. Of the prediction equations evaluated, a prediction model (Equation 13, Table 3 and Table 4) by Yan et al. [18] developed using beef cattle had the lowest RMSPE of 14.0% and highest *CCC* of 0.67. The equation developed in the present study across species (Equation (4)) had the same RMSPE of 14.0% and similar *CCC* of 0.66. Some of the errors associated with assessed model predictions may have been caused by using estimates for DOMD from measured ME values for cattle, accuracy of feed and CH_4_ measurements from different institutes and years, the random effect of experiment and the estimated effect of feeding level independent of diet components. Some of the limitations of the data used in the current study were that the sheep were fed a fixed amount (maintenance intake), whereas the cattle were offered an amount of feed based on their production level. The data collated also included similar numbers of records for individual sheep (*n* = 288) and dairy cows (*n* = 284) but fewer records for beef cattle, which may have contributed to an over-prediction on average (about −1.1 g/kg DM intake; Figure 3) of CH_4_ yield for beef cattle compared to sheep and dairy cows (1.6 and 1.1 g/kg DM intake respectively). Although the data used for model development and validation were randomly selected with the process repeated five times, both sets of data were from the same experiments and therefore not considered truly independent. This may have enhanced the accuracy of predictions for the model developed in the current study compared to predictions using equations obtained from the literature. 

The equations obtained from the literature were developed using cattle, except for the equation by Blaxter and Clapperton [6], which included sheep in its development. These equations were recommended based on their ability to predict enteric CH_4_ emissions in other evaluation studies [2,3], and also their ability to predict emissions over a wide range of DM intakes [8], which resulted in the exclusion of several equations from the literature (e.g., by Ellis et al. [32] and Moe and Tyrrell [33] that were developed on a narrower range of feed intakes). When comparing RMSPE, proportion of MSPE, *CCC*, location (ν) and scale shift (μ) values for prediction equations evaluated in the present study, several linear and nonlinear equations had comparable predictive performances across sheep and cattle studied, which were Equations (4), (7) to (9) and (13). These prediction equations are based on input variables of energy content, fiber content, forage in the diet and DM intake of the diet. 

The effect of diet, i.e., amount of intake and composition, has been found to account for a large proportion of variation in enteric CH_4_ emissions from dairy cows [4,34]. Indeed, it has been shown [35] that DM intake, or even a prediction of intake [36], could be used as a proxy for selecting animals on CH_4_ emissions, since it explains a large proportion of the variation in emissions. The current study showed that in the Rowett Institute data, the measured ME values for paired sheep fed the same diet and amount of feed were highly correlated (*CCC* = 0.926), but considerable variation existed in CH_4_ yield between sheep (*CCC* = 0.218) at the Rowett, as well as notable residual variation between observed and predicted CH_4_ yield using Equation (4) developed in the current study for sheep and cattle (Figure 3). This suggests there is variation in CH_4_ yield between animals that would allow genetic selection of low emitting animals. The observed mean CH_4_ yields in the current study were slightly higher (ranging from 22.6 for dairy cows to 29.3 g/kg DM intake for sheep) than those reported in other studies (range 14.9 to 23.7 g/kg DM intake) in which respiration chambers have been used to measure CH_4_ emissions from beef and dairy cattle [4,32,37,38]. The coefficient of variation between animals (ranged from 10% for beef cattle to 15% for dairy cows) was within the range of 3% to 34% in coefficient of variation between animals found in other studies [4,39]. Grandl et al. [40] found that CH_4_ yield changed in dairy cattle with age and was associated with changes in efficiency of fiber digestibility with age. In contrast, Ramirez-Restrepo [38] found no effect of age of animal on CH_4_ yield in dairy cattle on the same diet, which may be explained by older animals being included in the study by Grandl et al. [40]. Differences seen in the current study were attributed to diet composition, feeding level (maintenance versus production level) and genetic differences between animals plus random variation in experiments. 

In the current study, after daily CH_4_ emissions were adjusted for intake and the random effect of study, the diet components that had a significant effect on CH_4_ yield were digestible organic matter (DOMD), EE (oil) and feeding level (i.e., ME intake as multiples of maintenance requirement). Individual feeds can vary considerably in their effect on CH_4_ based on their chemical composition. For example, it has been suggested that undigested organic matter may influence rate of digestion [41], but the increased intake of less digestible feeds such as forage has little effect on CH_4_ yield [6]. Passage rate of substrate and rumen fluid dilution rate (influencing the ratio of acetate to propionate) have been found to explain 28% and 25%, respectively, of variation in CH_4_ production [42]. Reduced retention time of feed in the rumen may occur when the diet is highly fermentable, resulting in a reduction in CH_4_ production. More digestible and higher quality feed such as concentrate added to forage can increase post-ruminal digestion, particularly in the small intestine, which is energetically more efficient with lower CH_4_ losses than digestion in the rumen [43]. Diets become more metabolizable with increasing amounts of cereal grain in the diet, which has a curvilinear relationship with fibre digestion in mixed rations and results in a depression in CH_4_ yield [25], hence the nonlinear relationship used to estimate DOMD from ME content in the current study. The amount and type of dietary carbohydrate fermented affects rumen retention time of substrate, fermentation rate, and rates of production of acetate, propionate and hydrogen. More detailed measurements on fibre digestibility may have improved CH_4_ yield prediction and help describe the effect of feeding level seen in the current study. The prediction equations of Blaxter and Clapperton [6] and Yan et al. [26] adjusted CH_4_ for ME intake as multiples of maintenance energy requirement. This is based on the theory that a higher intake increases the fractional passage rate of feed through the rumen and reduces retention time, rumen digestion (dependent on the diet) and CH_4_ yield [42,44,45]. The average feeding level among sites (ranging from 1 to 3.7; Table 1 and Table 2) was consistent with the change in organic matter digestibility found by Nousiainen et al. [46] of 3.2 g/kg DM intake when supplementing a diet with concentrate feed. The effect of feed intake on CH_4_ yield from cattle in the current study was accounted for by DOMD estimated from ME content and ME intake as multiples of maintenance energy requirement. The effect of intake or feeding level on enteric CH_4_ has been included in prediction equations as ME intake as a multiple of maintenance energy required by the animal [6,26] as mentioned above, and as DM intake [4,32] or dry matter intake per kg of body weight [7]. In the present study, the feeding level variable was expressed as multiples of ME intake above maintenance intake i.e., the effect of feeding level on enteric CH_4_ was zero at the maintenance intake level, as found by Yan et al. [26]. In animals fed above maintenance, there was a negative response (−2.47) in CH_4_ yield with increasing feeding level, which is consistent with the feeding level response in Equation (7) of −2.453.

This research identified diet components that have significant effects on enteric CH_4_ yield from ruminant livestock and developed a prediction model based on these components. The model that best described CH_4_ yield included the diet components of DOMD, EE and feeding level. The current study found no significant effect of livestock type (sheep, beef cattle or dairy cows) or significant interaction between livestock type and diet component. Excluding the effect of feeding level, the significant effects of diet components were found to be consistent across species in the current study, whether fed at maintenance or production intake levels. This finding is consistent with the finding of Charmley et al. [31], who also found that common coefficients can be used to predict the CH_4_ yield of beef cattle and dairy cows. Ramin and Huhtanen [7] found similar important diet components and responses of similar magnitude to those of the present study (Equation (4)), with a positive response for organic matter digestibility at maintenance (0.076 kJ CH_4_/g) and negative response for EE (−0.13 kJ CH_4_/g). The response in CH_4_ yield of −0.113 g per gram increase in EE concentration in the current study was similar to the response found by Ramin and Huhtanen [7] and the response (−0.09) found by Moate et al. [37] for lactating dairy cows. In the study of Ramin and Huhtanen [7], the response of DM intake per kg of body weight (−0.70 kJ CH_4_/g) was also negative and similar to the effect of feeding level in the present study (assuming a 600 kg cow fed to its maintenance requirement of 60 MJ a diet of 11 MJ ME/kg DM and 18.5 MJ GE/kg DM, would consume 9.1 g DM intake/kg of body weight, which gives a response of (−0.7 × 9.1 × 18.5)/55.65 = −2.12 g CH_4_ per feeding level). In addition to these components, Ramin and Huhtanen [7] found NFC and neutral detergent fibre to be important variables, which was not observed in the current study as the effect of carbohydrate components was accounted for by inclusion of DOMD in the model. As discussed above, reductions in enteric CH_4_ yield appear possible by mechanisms that promote the passage of organic matter to post-rumen digestion and reduce rumen fermentation by high intakes of digestible feed and addition of fats.

## 5. Conclusions

The current study collated detailed information and used a large dataset to obtain the response of CH_4_ yield to different diet components across sheep, cattle, and diets. An equation to predict CH_4_ yield using measurable diet components of DOMD, EE and the feeding level of livestock was developed. Increasing DOMD concentration in feed increases CH_4_ yield, but increasing dietary EE and the feeding level above maintenance intake reduces CH_4_ yield. The model developed in this study was able to predict CH_4_ yield of ruminants fed at different production levels in mixed rations. Compared to observed values, the model developed slightly over-predicted the CH_4_ yield of beef cattle and under-predicted the CH_4_ yield of sheep and dairy cows. This research developed a method of predicting CH_4_ yield from feed using diet components that can be obtained from feed analysis information, which can be used in diet formulations to assess the effect of diet manipulation on GHG emissions.

## Figures and Tables

**Table 1:** Average production values and composition of diets fed to sheep at the Rowett Institute [13], beef cattle and dairy cows at AFBI and dairy cows at Ellinbank Research Centre for data used to develop CH_4_ prediction equations.

Component	Rowett Sheep		AFBI Beef Cattle		AFBI Dairy Cows		Ellinbank Dairy Cows		Pearson Correlation Coefficient ^3^	
	Mean ± s.d.	Range	Mean ± s.d.	Range	Mean ± s.d.	Range	Mean ± s.d.	Range	*r*	*p* Value
*Observations*	*n = 192*		*n = 47*		*n = 148*		*n = 41*			
Proportion forage	0.51 ± 0.20	0.25–0.75	0.81 ± 0.23	0.30–1	0.56 ± 0.22	0.27–1	0.86 ± 0.14	0.64–1		
Dry matter intake, kg/day	0.9	-	7.0 ± 1.1	5.0–10.1	17.1 ± 3.4	7.9–24.5	15.7 ± 2.2	12.0–19.6		
Milk yield, kg/day	-	-	-	-	23.9 ± 8.1	5.2–46.8	15.3 ± 5.6	4.7–30.5		
Live weight, kg	-	-	499 ± 57	372–617	571 ± 61	385–713	560 ± 63	441–684		
DOMD ^1^, g/kg DM	684 ± 47	564–787	720 ± 29	654–768	741 ± 26	657–811	703 ± 46	579–804	0.093	0.224
Lignin, g/kg DM	39.7 ± 9.5	16.8–76.2	-	-	-	-	42.2 ± 7.9	28.6–59.0	−0.161	0.015
Starch, g/kg DM	156 ± 77	38–313	67.0 ± 79.7	0–242	101 ± 50.3	0–168	102 ± 86	1.9–230	0.163	0.015
Sugar, g/kg DM	35.2 ± 16.1	11.8–74.1	34.3 ± 8.0	20–51.6	46.6 ± 13.2	20–63.9	98.1 ± 34.8	55.9–193	−0.483	<0.001
Neutral detergent fibre, g/kg DM	427 ± 92	235–594	515 ± 74	359–649	417 ± 75	266–583	446 ± 55	356–536	−0.070	0.299
Acid detergent fibre, g/kg DM	266 ± 60	137–369	299 ± 55	169–374	248 ± 45	170–362	278 ± 43	197–352	−0.023	0.733
Crude protein, g/kg DM	137 ± 22	91.8–190	145 ± 13	120–160	183 ± 21	130–245	185 ± 30	128–251	−0.341	<0.001
Ash, g/kg DM	74.9 ± 14.8	48.8–126	77.4 ± 17.6	43.2–105	84.9 ± 8.8	57.2–111	89.6 ± 15.9	63.5–121	−0.209	0.005
Ether extract (oil), g/kg DM	33.1 ± 9.9	16.5–64.4	38.3 ± 3.2	31.4–44.0	55.4 ± 5.7	44.0–63.0	33.6 ± 6.6	26.0–53.0	−0.086	0.215
Feeding level ^2^	1	-	1.6 ± 0.2	1.3–2.3	3.7 ± 0.7	1.7–6.1	3.2 ± 0.7	1.9–4.5	−0.560	<0.001
Gross energy, MJ/kg DM	18.4 ± 0.4	17.0–19.4	18.5 ± 0.5	17.4–19.7	18.6 ± 0.5	17.2–19.8	18.5 ± 0.9	16.8–20.4	−0.050	0.529
Digestible energy, MJ/kg DM	13.1 ± 1.1	10.5–15.6	13.8 ± 0.8	12.2–15.1	14.2 ± 0.7	12.7–16.6	13.3 ± 1.0	10.6–15.4	0.106	0.169
Metabolizable energy, MJ/kg DM	10.8 ± 1.0	8.6–13.4	11.6 ± 0.7	10.1–12.8	12.1 ± 0.7	10.2–14.1	11.2 ± 1.1	8.6–13.9	−0.114	0.129
Methane production, g/day	25.7 ± 3.2	17.6–34.6	183 ± 30	110–246	379 ± 67	208–539	366 ± 64	262–495		
Methane yield, g/kg DM intake	29.4 ± 3.6	20.2–39.5	26.2 ± 2.5	18.9–33.3	22.6 ± 3.5	14.4–32.9	23.4 ± 2.8	16.3–28.4		

^1^ digestible organic matter content (DOMD) was calculated by multiplying organic matter content of the feed by digestibility of organic matter (OMD) at the Rowett. At AFBI and Ellinbank, DOMD was estimated from data in Third Report of Rowett Feedingstuffs Evaluation Unit [13] as: DOMD (g/kg DM) = 472.49 × ln (ME) − 437.69; ^2^ expressed as ME intake as multiples of animal maintenance energy requirement calculated from AFRC [17]; ^3^ Pearson correlation coefficient (*r*) for association between diet components and CH_4_ yield.

**Table 2:** Average production values and composition of diets fed to sheep at the Rowett Institute [13], beef cattle and dairy cows at AFBI and dairy cows at Ellinbank Research Centre for data used to evaluate CH_4_ prediction equations.

Component	Rowett Sheep		AFBI Beef Cattle		AFBI Dairy Cows		Ellinbank Dairy Cows	
	Mean ± s.d.	Range	Mean ± s.d.	Range	Mean ± s.d.	Range	Mean ± s.d.	Range
*Observations*	*n = 96*		*n = 24*		*n = 74*		*n = 21*	
Proportion forage	0.49 ± 0.21	0.25–0.75	0.81 ± 0.22	0.34–1	0.54 ± 0.21	0.28–1	0.85 ± 0.14	0.66–1
Dry matter intake, kg/day	0.9	-	6.8 ± 1.2	4.9–10.0	17.2 ± 3.3	8.1–23.9	15.6 ± 2.1	12.2–19.0
Milk yield, kg/day	-	-	-	-	24.1 ± 8.2	4.5–45.7	15.6 ± 6.1	6.0–30.9
Live weight, kg	-	-	495 ± 57	381–601	572 ± 58	432–728	550 ± 64	422–670
DOMD ^1^, g/kg DM	682 ± 47	570–781	722 ± 29	665–781	741 ± 27	665–794	704 ± 51	588–807
Lignin, g/kg DM	40.8 ± 10.3	19.0–74.6	-	-	-	-	42.0 ± 8.3	30.1–56.8
Starch, g/kg DM	156 ± 75	40–311	66.8 ± 77.4	0–228	104 ± 48	0–166	111 ± 86	6.0–225
Sugar, g/kg DM	37.1 ± 17.6	11.9–74.1	34.3 ± 7.9	20–49.9	47.4 ± 12.7	20–63.2	96.3 ± 33.9	57.1–174
Neutral detergent fibre, g/kg DM	424 ± 91	249–588	515 ± 68	373–633	413 ± 74	265–583	441 ± 58	351–553
Acid detergent fibre, g/kg DM	265 ± 60	146–365	300 ± 52	189–373	247 ± 47	170–360	276 ± 46	213–349
Crude protein, g/kg DM	138 ± 21	93.0–189	146 ± 12	121–160	184 ± 22	130–245	179 ± 28	129–231
Ash, g/kg DM	75.9 ± 14.9	50.6–124	78.2 ± 17.9	44.6–105	83.9 ± 8.1	59.6–110	86.1 ± 13.8	65.4–113
Ether extract (oil), g/kg DM	33.9 ± 10.3	16.8–63.9	38.3 ± 3.1	32.0–44.0	56.0 ± 5.5	44.0–62.8	32.5 ± 5.5	25.5–45.9
Feeding level ^2^	1	-	1.5 ± 0.2	1.3–2.2	3.7 ± 0.7	1.8–5.8	3.2 ± 0.6	2.1–4.5
Gross energy, MJ/kg DM	18.4 ± 0.4	17.1–19.3	18.6 ± 0.5	17.5–19.5	18.6 ± 0.4	17.3–19.6	18.6 ± 0.9	17.1–20.3
Digestible energy, MJ/kg DM	13.1 ± 1.0	10.7–15.7	13.9 ± 0.8	12.4–15.2	14.2 ± 0.7	12.8–15.8	13.3 ± 1.1	11.1–15.7
Metabolizable energy, MJ/kg DM	10.7 ± 0.9	8.7–13.5	11.7 ± 0.7	10.3–13.2	12.1 ± 0.7	10.3–13.6	11.3 ± 1.2	8.7–14.0
Methane production, g/day	25.5 ± 3.1	17.6–34.0	179 ± 31	117–240	378 ± 68	211–528	363 ± 64	248–487
Methane yield, g/kg DM intake	29.1 ± 3.5	20.2–38.8	26.5 ± 2.6	21.8–32.8	22.3 ± 3.4	14.8–30.1	23.5 ± 3.3	15.0–27.8

^1^ digestible organic matter content (DOMD) was calculated by multiplying organic matter content of the feed by digestibility of organic matter (OMD) at the Rowett. At AFBI and Ellinbank, DOMD was estimated from data in Third Report of Rowett Feedingstuffs Evaluation Unit [13] as: DOMD (g/kg DM) = 472.49 × ln (ME) − 437.69; ^2^ expressed as ME intake as multiples of animal maintenance energy requirement calculated from AFRC [17].

**Table 3:** Selected enteric CH_4_ prediction equations from the literature evaluated in present study with emissions expressed per kilogram of DM intake.

Reference	Equation No.		Equations ^1^
[29]	5	CH_4_ (g/kg DMI)	=(18 + 22.5 × DMI)/DMI
[6]	6		=(1.3 + 11.2 × DE/GE + FL × (2.37 − 5 × DE/GE)/100 × GE × DMI/0.05565/DMI
[26]	7		=(DE × DMI × (0.094 + 0.028 × (FADF/ADF × DMI)) − 2.453 × (FL − 1))/0.05565/DMI
[26]	8		=(DE × DMI × (0.096 + 0.035 × (FDMI/DMI)) − 2.298 × (FL − 1))/0.05565/DMI
[4]	9		=(56.27 − (56.27 + 0) × e^(−0.028 × DMI)^)/0.05565/DMI
[4]	10		=(45.89 − (45.89 + 0) × e^(−0.003 × ME × intake)^)/0.05565/DMI
[30]	11		=(74.43 − (74.43 + 0) × e^(−0.0163 × DMI)^)/0.05565/DMI
[30]	12		=(7.16 − 0.101 × DMI)/100 × GE × intake/0.05565/DMI
[18]	13		=((0.877 − 14.66 × ME/GE + 13.55 × DE/GE + 0.457 × FDMI/DMI + 4.153 × NDF/1000 − 7.47 × ADF/1000) × GE × DMI + 0.8) × 0.003954/0.05565/DMI

^1^ DMI = dry matter intake (kg/day); DE = digestible energy (MJ/kg DM); ME = metabolizable energy (MJ/kg DM); GE = gross energy (MJ/kg DM); FL = multiples of ME intake over maintenance calculated from AFRC (1993); NFC = non-fibre carbohydrate (kg/day); HC = hemicellulose (kg/day); C = cellulose (kg/day); ADF = acid detergent fibre (g/kg DM); FADF = forage ADF (kg/day); FDMI = forage DMI (kg/day); NDF = neutral detergent fibre (g/kg DM).

**Table 4:** Evaluation of prediction equations for CH_4_ emissions developed in the present study and developed elsewhere (Table 3), using data from sheep (*n* = 96), beef (*n* = 24) and dairy cattle (*n* = 95).

Equation	CH_4_ ± s.e. (g/kg DM)		RMSPE % ^2^	Proportion of MSPE			Lin’s Concordance				
	Predicted ^1^	Actual		ER ^2^	ECT ^2^	ED ^2^	*r* ^3^	*C_b_* ^4^	*CCC* ^4^	ν	μ
4—sheep	27.5 ± 0.2	29.1 ± 0.4	13.7	0.19	0.03	0.78	0.456	0.706	0.321	0.62	1.93
4—beef	27.5 ± 0.3	26.5 ± 0.5	11.1	0.12	0.01	0.87	−0.044	0.687	−0.017	−0.53	2.05
4—dairy	21.6 ± 0.2	22.6 ± 0.3	15.4	0.06	0.33	0.61	0.413	0.835	0.303	0.37	1.62
4—across species	24.9 ± 0.2	25.9 ± 0.3	14.0	0.08	0.10	0.82	0.696	0.940	0.655	0.25	1.30
13	25.3 ± 0.3		14.0	0.02	0.15	0.82	0.690	0.974	0.673	0.14	1.21
9	23.5 ± 0.2		15.3	0.36	0.01	0.62	0.671	0.889	0.597	0.64	1.52
7	25.4 ± 0.2		15.3	0.02	0.15	0.83	0.608	0.785	0.477	0.16	1.75
8	24.4 ± 0.2		17.4	0.12	0.16	0.73	0.543	0.926	0.502	0.43	1.66
10	25.2 ± 0.2		20.0	0.02	0.37	0.61	0.473	0.842	0.398	0.19	1.48
12	20.9 ± 0.2		24.1	0.64	0.07	0.24	0.664	0.469	0.312	1.44	1.77
6	21.7 ± 0.4		24.7	0.44	0.32	0.24	0.703	0.740	0.521	0.81	0.76
11	20.4 ± 0.1		25.5	0.70	0.07	0.24	0.550	0.297	0.163	2.29	3.64
5	32.5 ± 0.7		65.7	0.15	0.81	0.04	0.559	0.542	0.303	−0.99	0.48

^1^ The CH_4_ yield was predicted using the following equation: CH_4_ (g/kg DM intake) = 0.046 × DOMD − 0.113 × EE (both g/kg DM) − 2.47 × (feeding level − 1). The DOMD is the digestible organic matter; ^2^ root mean square prediction error (RMSPE) expressed as a percentage of the observed mean, with proportions of total MSPE due to mean bias (ER), line bias (ECT) and random variation of the regression slope (ED); ^3^ Pearson correlation coefficient (*r*); ^4^ Lin’s concordance analysis with bias correction factor (*C_b_*), concordance correlation coefficient (*CCC*), location shift (ν) and scale shift (μ) values.
